# Is there a link between childhood adversity, attachment style and Scotland’s excess mortality? Evidence, challenges and potential research

**DOI:** 10.1186/s12889-016-3201-z

**Published:** 2016-07-28

**Authors:** M. Smith, A. E. Williamson, D. Walsh, G. McCartney

**Affiliations:** 1 NHS Greater Glasgow and Clyde, Commonwealth House, 32 Albion Street, Glasgow, G1 1LH UK; 2 General Practice and Primary Care, School of Medicine, University of Glasgow, 1 Horselethill Road, Glasgow, G12 9LX UK; 3 Glasgow Centre for Population Health, Olympia Building, 2-16 Orr Street, Bridgeton Cross, Glasgow, G40 2QH UK; 4 NHS Health Scotland, Meridian Court, 5 Cadogan Street, Glasgow, G2 6QE UK

**Keywords:** Scotland, Public health, Mortality, Health inequalities, Social determinants of health, Adverse childhood experiences, Attachment

## Abstract

**Background:**

Scotland has a persistently high mortality rate that is not solely due to the effects of socio-economic deprivation. This “excess” mortality is observed across the entire country, but is greatest in and around the post-industrial conurbation of West Central Scotland. Despite systematic investigation, the causes of the excess mortality remain the subject of ongoing debate.

**Discussion:**

Attachment processes are a fundamental part of human development, and have a profound influence on adult personality and behaviour, especially in response to stressors. Many studies have also shown that childhood adversity is correlated with adult morbidity and mortality.

The interplay between childhood adversity and attachment is complex and not fully elucidated, but will include socio-economic, intergenerational and psychological factors. Importantly, some adverse health outcomes for parents (such as problem substance use or suicide) will simultaneously act as risk factors for their children.

Data show that some forms of “household dysfunction” relating to childhood adversity are more prevalent in Scotland: such problems include parental problem substance use, rates of imprisonment, rates of suicide and rates of children being taken into care. However other measures of childhood or family wellbeing have not been found to be substantially different in Scotland compared to England.

**Summary:**

We suggest in this paper that the role of childhood adversity and attachment experience merits further investigation as a plausible mechanism influencing health in Scotland. A model is proposed which sets out some of the interactions between the factors of interest, and we propose parameters for the types of study which would be required to evaluate the validity of the model.

## Background

### Scotland’s excess mortality: alcohol, drugs, suicide and violence

Since about 1950, improvements in health in Scotland have not kept pace with those in other countries [1]. Between 1950 and 1980, higher mortality in Scotland compared to the rest of Europe was largely attributable to cardiovascular disease, stroke and cancer [2]. Since about 1980, these higher death rates from physical causes have been joined by higher mortality rates from alcohol, drugs, suicide and violence, especially in Glasgow and the West Central Scotland conurbation.

These higher mortality rates relative to the rest of the UK persist even after adjustment for differences in area deprivation and individual social class, and so have been termed “excess” mortality [3].

In the mid to late 2000s mortality at all ages was about 15 % higher in Glasgow compared to Liverpool and Manchester, similar cities in England. But premature deaths (in people less than 65 years old) were 30 % higher, and the causes of those deaths were different. Well over half of the excess premature mortality was attributable to deaths from alcohol, drugs-related poisonings, suicide and “external causes”, which includes violence [4]. Current estimates of the excess are of a 10 % higher all-age mortality and a 20 % higher premature mortality in Scotland compared to England and Wales. That excess would account for an extra 5,000 deaths per year in Scotland [5].

Why does West Central Scotland have high rates of problem substance use, suicide and violence, even after deprivation and other conventional aetiological factors have been controlled for? Epidemiological studies have not been able to fully explain the causes of this excess mortality. Researchers have systematically examined a range of potential explanatory factors (including inequality, the effects of migration, genetic differences, parenting style, domestic violence, family disruption, maternal mental health, sectarianism and climatic influences) and not found convincing effects [6–8]. Some authors have argued that these health and social problems reflect the impact of neoliberal economic policies [9] as part of a broader “political attack” [6]. The most recent research has pointed to a greater vulnerability in the Scottish population caused by a series of historical processes and decisions [10].

Harmful early years and childhood experiences have also been proposed as a possible explanation for the excess of poor health and mortality seen in Scotland [8, 11]. This paper describes the main approaches to understanding childhood influences on adult health, proposes a model for their interaction at a population level, and reviews the issue of excess mortality in Scotland in the light of current data. The implications for future research are discussed.

### Conceptualising the impact of negative experiences

We have long known that childhood experiences can affect health in adulthood. There are five main concepts that currently shape our understanding of those influences.

#### Adverse childhood experiences

The landmark Adverse Childhood Experiences (“ACE”) studies by Felitti et al. used a retrospective epidemiological approach to ask research participants about exposure to a range of harmful or distressing experiences before the age of 18 years. Those experiences included domestic violence, physical or sexual abuse, emotional neglect, parental separation, alcohol and drugs misuse; and mental illness, suicide or imprisonment affecting a member of the household in which the child grew up. These experiences may be categorised and counted using a 10-item questionnaire [12].

Two thirds of the initial ACE study population (all adult members of a Health Maintenance Organisation in the USA) reported an score of one or more, and 12.5 % a score of four or more [13].

Felitti et al’s study and similar work conducted more recently in the UK [14–16], show a clear correlation between childhood adversity and physical and mental health problems in adulthood. A number of well-designed prospective studies have confirmed that the overall burden of childhood adversity is correlated with poor outcomes [17].

**Fig. 1:**
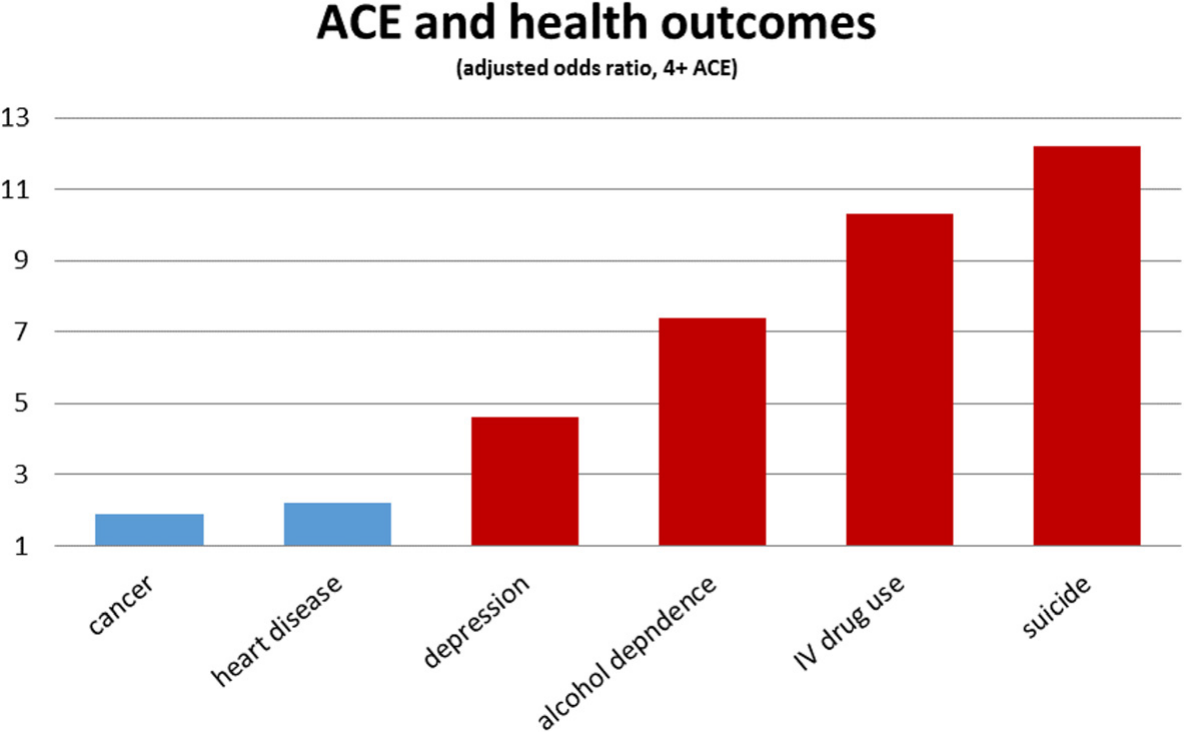
Association of childhood adversity with some adult physical health problems (shown in *blue*) and mental health and addiction problems (shown in *red*). The graph is adapted from Felitti [12], & shows odds ratios adjusted for age, gender, race, and educational attainment for adults exposed to four or more Adverse Childhood Experiences (ACE)

Figure 1 shows that exposure to four or more categories of adverse childhood experiences in the original ACE study increased the risk of adult physical illnesses such as cancer and heart disease about two-fold; but the increased risk of mental or behavioural problems was between 4- and 12-fold (after adjustment for age, gender, race, and educational attainment).

Exposure to such childhood adversity is common: for example, 9·6 % of children in a European community survey reported sexual abuse, 22·9 % physical abuse and 29·1 % mental abuse [18].

In UK studies, increasing exposure to childhood adversity has also been strongly related to adverse behavioural, mental and physical outcomes. There were no significant relationships between socio-economic (SE) deprivation and having between one and three ACEs, yet having four or more ACEs was strongly associated with higher SE deprivation [15].

Studies consistently show strong, graded associations between childhood adversity and the risk of adult problem drug [19] and alcohol [20] use, and with the development of anxiety and depression in adults [21–23]. Childhood adversity is associated with a large increase in suicide risk [24, 25]. It is estimated that between one fifth and one third of adult mental health problems are attributable to childhood adversity [24], and adversity seems to aggravate the functional impairment caused by such difficulties [26].

Exposure to childhood adversity is associated with lack of empathy, impulsivity and anger, and the emergence of behavioural problems [27], including delinquency [28, 29] and social alienation [30].

Each child will respond individually to their situation, and a minority of children may develop positively despite severe adversity [31]. Protective relationships with adults facilitate a child’s response to adversity, and are critical to healthy development [32].

#### Complex trauma

Complex trauma (also known as “complex post-traumatic stress disorder” or “type 2” trauma) is a term that describes psychological harm arising from repeated or prolonged trauma, including abuse or neglect during childhood. First described by Herman in the early 1990s [33], the concept has a growing influence on clinical work in psychology, the addictions field and homelessness health.

Stressors leading to complex trauma are repetitive or prolonged, involve direct harm or neglect by caregivers, occur at developmentally vulnerable times, and compromise child development and adult functioning. This leads to patterns of thinking, feeling and behaving across a person’s life course which are characterised by impulsiveness, emotional lability, difficulty functioning in relationships and symptoms of Post-Traumatic Stress Disorder.

These difficulties mean that people with complex trauma backgrounds are more likely to be involved in health harming behaviours and be exposed to ongoing risky relationships, violence and re-victimisation [34]. Complex trauma can be a practical explanatory framework for understanding the impact of childhood adversity on adult functioning and behaviour, and the use of “trauma informed practice” principles can assist in the development of public services and individual professional client relationships to better meet adults’ needs [35].

Standard interview schedules used to measure complex trauma include items ranging from “having someone close die unexpectedly”, to “saw atrocities/carnage” [36], and therefore contain a wider range of experiences than the ACE measures described above. Studies suggest that childhood trauma is highly prevalent [37], especially amongst adults who experience problem substance use [38, 39].

Criteria have been proposed for a diagnosis of complex trauma [34], but this was not included in the updated Diagnostic and Statistical Manual of Mental Disorders (DSM-5) in 2013 [40].

#### Attachment

Attachment theory was developed by the psychoanalyst John Bowlby on the basis of observations of young children’s behaviour when separated from their parents. In order to survive, infants have to be able to form a bond with their caregiver (usually the mother), and to elicit a care-giving response from her. This “affectional bond” with a “differentiated and preferred individual” [41] gives the child a “safe haven” to return to at times of stress, and a “secure base” from which to explore the world and gain independence.

As children interact with caregivers during infancy and childhood, they develop an “internal working model” of themselves in relation to others [42]. This model helps children to predict and understand others’ responses, to make sense of the emotions associated with interpersonal experiences, and is therefore a fundamental aspect of personality development as the child grows up.

Bowlby’s ideas about child development have been endorsed and extended by decades of empirical evidence [43].

When carers are sensitive and responsive to a child’s needs, a secure attachment style develops. Children who are securely attached tend to have a positive self-image, a capacity to manage distress, and the ability both to function independently and also to interact with others [44]. This is the case for the majority of children.

By contrast, insecurely-attached individuals tend to respond to stress with anxious features, including exaggerated emotional responses; or with avoidant characteristics, including suppressing negative feelings and withdrawal from help-seeking contact [45].

These attachment patterns have a profound impact, not only on childhood development but also on adolescent and adult personality and relationships, including romantic relationships and choice of partner [46–49]. Attachment style may have a strong influence on children’s ability to learn [50, 51].

Securely-attached parents tend to raise securely attached children; similarly, parents with a fearful, avoidant or disorganised attachment experience tend to raise children with a similar attachment style [52]. The mechanisms for such inter-generational effects are complex, but probably mediated in part by the quality of family relationships and parental psychosocial functioning [53, 54].

The problems associated with dysfunctional attachment experiences are most evident when security is threatened [55]. This is readily seen in children who are tired, sick or frightened; but can also be seen in adults exposed to stressful situations [55–58]. Adults with an attachment style that enables trusting and emotionally supportive relationships may be less vulnerable to depression and physical illness [59–61].

#### Allostatic load

Human stress responses are mediated by hormones which are essential for adaptation, metabolic stability, and survival. But longer-term exposure to fluctuating or heightened stress incurs a physiological cost, which has been termed “allostatic load”. It has been demonstrated that this load can accelerate disease processes [62], and may lead to harmful physical, behavioural and cognitive effects [63] which begin in the prenatal period and may continue through the life span. Children exposed to adversity show markers of immune and metabolic change consistent with the abnormal functioning of these stress-sensitive systems [64]. Such changes may be sensitive to developmental stage, and are modified by experiences of care-giving from adults [63]. Parental exposure to trauma during childhood is related to deficits in social-emotional development in their children [65]. Lower socio-economic status is associated with increased allostatic load [66].

#### Toxic stress

The concept of “toxic stress” seeks to synthesise the evidence from work on allostatic load, adverse childhood experiences and attachment theory. It arises from the strong, frequent, or prolonged activation of the body’s stress response systems in the absence of the buffering protection of a supportive, adult relationship [32]. Unlike allostatic load, which reflects the burden of stress alone, “toxic stress” considers the balance between stress and protective factors.

Toxic stress compromises children’s ability to cope [67], and increases the risk of future mental and physical health problems [68, 69], perhaps through epigenetic effects [70].

It is hypothesised that the extent to which stress might be “tolerable” or “positive” rather than “toxic” will depend on genetic predispositions and the availability of supportive relationships, including secure attachment experiences [32].

### Modelling a relationship between childhood experience of adversity and adult health outcomes

Some of the issues described in the previous section overlap; Table 1 summarises their characteristics and seeks to clarify their inter-relationships. For the purposes of this paper, these approaches to understanding childhood experience can be summarised as:

- exposure to potentially harmful experiences (characterised as ACE for children, and as “complex trauma” for a wider range of experiences in children and adults)
- the response to those experiences as influenced by attachment style and psychological development: securely-attached children (and parents) are likely to respond adaptively to stress, whereas insecure or disorganised attachment styles often predispose to further stress.
- the consequences of those experiences, in terms of allostatic load, toxic stress, psychological development and adult attachment style

**Table 1:** Concepts relating childhood experience to adult outcomes

		Definitions
Exposure	Adverse childhood Experiences	An epidemiological measure of childhood abuse, neglect, and family dysfunction, which can be assessed in adults using a ten-item Adverse Childhood Experiences (ACE) questionnaire. ACEs include emotional and physical abuse or neglect, sexual abuse, and exposure to household violence or substance misuse. ACE would include most forms of childhood trauma.
	Complex Trauma	A psychological construct that relates childhood traumatic experiences to adult emotions and behaviour. A particular focus on health harming behaviours, and on service responses to those problems.
Response	Attachment	A fundamental aspect of human development: infants’ biological instinct to develop a relationship with at least one caregiver for safety and protection. Over time, the “attunement” developed in such relationships helps the child to regulate their feelings and make sense of the world. Secure attachment develops when parents consistently respond to their child’s needs. Patterns of “insecure” attachment include resistant, avoidant and disorganised types. Attachment theory has also been applied to adult relationships.
Consequences	Allostatic Load	The physiological consequences of chronic exposure to fluctuating or heightened stress, which may lead to physical, behavioural and cognitive effects.
	Toxic stress	Prolonged activation of the body’s stress response, occurring when a child experiences strong, frequent, and/or enduring adversity without the protection of a supportive adult relationship. Such adversity could arise from the burden of longstanding poverty, and the forms of ACE described above. Integrates aspects of both exposure and “resilience” to traumatic experiences.

These concepts are distinct but overlap, and have a dynamic interaction over the lifecourse. The relationship between the experience of adversity and attachment style is particularly important, since each may influence the other.

For example, children living in poverty are more likely not only to experience more adversity [16, 71], but also to develop insecure attachments, and this may be mediated through mothers’ care-giving behaviour [72, 73].

Poor outcomes in relation to childhood adversity are strongly linked to less stable family structures [74]. Furthermore, negative life events (such as loss of a sibling, marital breakdown, or severe illness) are not only causes of adversity in their own right, but may also threaten the quality of attachment relationships [75–77].

**Fig. 2:**
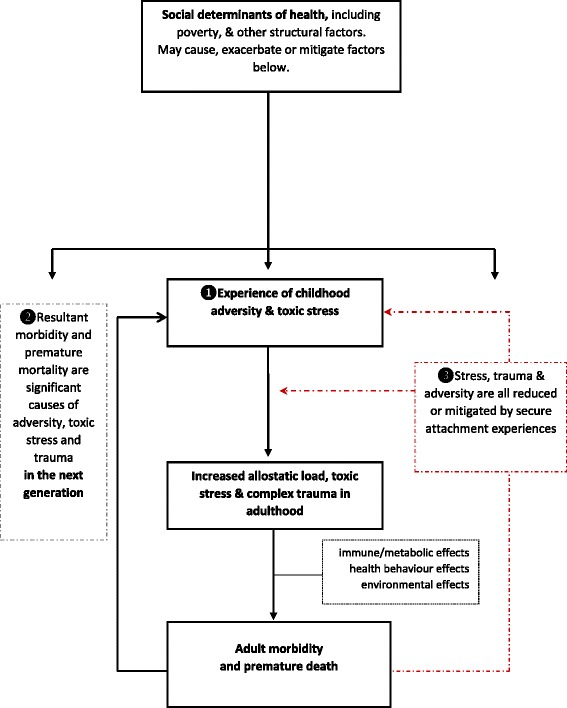
Model linking childhood stress, trauma and adversity to adult health outcomes, showing the modifying effect of attachment experience

Figure 2 outlines a model relating childhood adversity, complex trauma, attachment style, allostatic load and toxic stress complex trauma to excess mortality.

There are three main elements to the model. Negative socio-economic and “structural” factors predispose to childhood adversity and toxic stress (1). This causes increased allostatic load and toxic stress into adulthood, which in turn leads to premature mortality mediated by metabolic, immune, behavioural and environmental pathways.

Some forms of premature morbidity and mortality in parents will also act as adverse childhood experiences for their children (2), thereby creating the potential for inter-generational transmission of adversity.

Finally, attachment-related experiences and behaviours may act to mitigate the effects of adverse and stressful experiences (3).

In this model, differing socio-economic contexts can be the cause of adverse experiences, or act to exacerbate or mitigate the consequences of such experiences.

This model:

- is consistent with concepts of “critical periods” for childhood development, and of a cumulative burden of allostatic load through the lifespan [78].
- is consistent with structural and social models of health, but emphasises important non-socio-economic influences [78].
- links some adult outcomes to childhood risk factors, creating a potential feedback loop which could persist even if the initiating external socio-economic circumstances were to remit.
- argues that harm caused by exposure to ACEs and toxic stress can potentially be prevented or modified by secure attachment experiences.

The interaction between individuals, groups and their socio-economic environment is not entirely predictable, and since each will affect the other, a “complex adaptive system” emerges [79]. The timing and latency of the biological and social influences in Fig. 2 remains only partially understood. Nonetheless, we can expect that there will be interaction between “critical periods” for exposures in childhood, a cumulative burden of exposure through the lifespan, and a dynamic relationship with socio-economic circumstances. Each is important, and all interact [80].

### A political dimension to parenting and adversity

The extent to which individuals should be held responsible for their own health and behaviours is the subject of controversy and political debate. It is important to emphasise that this paper does not seek to add to the “long and undistinguished pedigree” of stereotypes about disadvantaged people in society [81].

Welshman summarises at least nine political “reconstructions” of social problems: the “social residuum” (1880s); the “unemployable” (1900s); the “social problem group” (1930s); the “problem family” (1950s); a “culture of poverty” (1960s); the “cycle of deprivation” (1970s); the “underclass” (1980s); “social exclusion” (1990s) and the idea of “troubled families” in Britain (from 2010) [82].

The model described within this paper would be inconsistent with any attempt to “blame the victim” for social and health problems, for the following three reasons.

First, every person has an experience of attachment, not only those living in poverty [83]; second, neither the adversity a child experiences nor the quality of their attachment is under that child’s control, and therefore cannot be their responsibility; and finally and most importantly, adversity and attachment experiences reflect a dynamic relationship between an individual, those close to them, and the way wider society is structured.

### Current evidence for the model and excess mortality in West Central Scotland

#### Adult morbidity and premature death

**Fig. 3:**
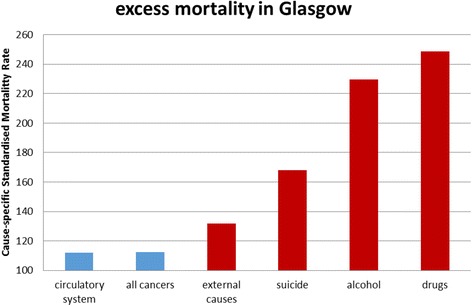
Cause-specific standardised mortality ratios (standardised by age, gender and area deprivation), 2003-07, for causes of death in Glasgow compared to Liverpool and Manchester (Liverpool and Manchester combined = 100). Mortality associated with physical health problems is shown in *blue*, and with mental health problems is shown in *red*. “External causes” includes deaths due to violence, which are not necessarily attributable to mental health problems. The graph is adapted from Walsh et al. [86]

Figure 3 shows the contribution of different types of physical and mental health problems to Glasgow’s excess mortality. A similar, though less marked, pattern can be observed in West Central Scotland, and in Scotland as a whole. The data show higher mortality from physical causes like cardiovascular disease and cancer, but the greatest relative differences are for “behavioural” causes: deaths due to external causes (including violence), suicide, alcohol and drugs. For premature mortality (deaths <65 years), half the ‘excess’ is attributable to alcohol and drugs, with a major contribution from suicide.

#### Experience of childhood adversity and toxic stress

Since violence, suicide and addictions are strongly correlated with adverse childhood experiences, what does current evidence tell us about that experience in Scotland compared to England, or in Glasgow compared to the similar cities of Liverpool or Manchester?

If premature mortality in Scotland and especially the West of Scotland were associated with differences in attachment experience or ACEs compared to England, then we would expect to see:

- Increased rates of “household dysfunction” in Scotland (eg substance misuse, imprisonment, mental illness) in households with children
- A higher prevalence of parenting indicators associated with insecure attachment (eg maternal stress or distress, or lack of paternal engagement)

Five data sources allow us to assess these predictions against data comparing the geographical areas of interest.

1. A comparison of “poverty, parenting and poor health” between Scotland and England, and the Glasgow & Clyde Valley, Merseyside and Greater Manchester. This study used a range of data sources but principally relied upon the four British Cohort Studies collating longitudinal data between 1946 and 2000 [8].
  This study found that the social and material circumstances experienced by people living in Scotland and Glasgow and the Clyde Valley (GCV) were broadly similar to those in England, Merseyside and Greater Manchester.
  However several measures of household dysfunction were higher in Scotland and Glasgow and the Clyde Valley compared to comparator areas in England.
  Problem opiate drug use was twice as common in Scotland compared to England.
  Rates of children being taken into care may act as a proxy measure of childhood adversity, and have been about twice as high in Scotland compared to England for the last three decades [8]. However legislative and practice differences between countries mean direct comparisons are difficult to make.
  Reports of actual and threatened domestic violence were high in the Glasgow and Clyde area compared to the English regions. Rates of imprisonment were also higher in the Glasgow and Clyde area compared with the English regions.
2. A between-country comparison of suicide rates in the UK showed that in the early to mid-2000s, Scotland’s national suicide rate was 79 % higher than in England, and twice as high for people aged 15 to 44 years [84]. In this context, suicide is relevant both as an adverse outcome (leading to premature mortality), but also as an adverse exposure – at least in families with children – as an indicator of “household dysfunction”).
3. Further analyses of data in the 1958 National Child Development Study [14] (one of the longitudinal sources included in the first study described above) specifically considered exposure to childhood adversity between Scotland and England, and between the Glasgow & Clyde Valley, Merseyside and Greater Manchester (Taulbut, personal communication). This showed a counterintuitive pattern of differences. The 1958 cohort study collected information about children’s experience of adversity. The items are listed included in Appendix 1, and included information about children being taken into care, living in a household where a family member was an offender, parental separation or loss, household mental illness, evidence of malnourishment as reported by a teacher, and presence of alcohol abuse in a family member as reported by a health visitor.
  The data show that children born in Scotland in 1958 may have been slightly more likely to experience two or more adversities in childhood compared to children born in England – 9.8 % (95 % CI 8.4–11.2 %) in Scotland compared to 7.9 % (95 % CI 7.4–8.4 %) in England. However, using the same measures, there were no meaningful differences between children born in Glasgow and the Clyde Valley (8.0 % (95 % CI 6.0–10.0 %), Merseyside (9.0 % (95 % CI 6.2–11.8 %) and Greater Manchester (12.3 % (95 % CI 9.1–15.5 %)).
  In other words, the data suggest that there may be slightly more exposure to childhood adversity in Scotland compared to England, but not necessarily among people born in Glasgow and the Clyde Valley in 1958 when compared with Scotland as a whole, and also to Merseyside and Greater Manchester.
  This is a counter-intuitive finding, since if exposure to childhood adversity were higher in Scotland than England, one would expect the difference to be most marked in the area of highest socio-economic deprivation with the highest subsequent mortality, i.e. Glasgow and the Clyde Valley.
4. An examination of the 1958 National Child Development Study and the 1970 British Cohort Study comparing childhood experiences in Scotland and England, and in Glasgow and the Clyde Valley compared to Merseyside or Greater Manchester [85].
  This study used different data from the 1958 National Child Development Study and the 1970 British Cohort Study to examine the relationship between childhood experiences and behavioural, respiratory and reading/vocabulary problems in children aged 5 and 7 years. The measures used in this study are summarised in Appendix 2. There was no evidence that early years experience was worse in Scotland compared to England, nor in Glasgow and the Clyde Valley compared to Merseyside or Greater Manchester. Although three of the items analysed in this study might relate to attachment experience (breastfeeding, being read to and “dad helping mum to put child to bed”), the indicators only included one of the 10 ACE factors recognised by Felitti (poor maternal mental health) [12]. These studies therefore needs to be interpreted with caution in this context.
5. A cross-sectional survey of the populations of Glasgow, Liverpool and Manchester carried out in 2011 examined adult psychological function and self-reported childhood happiness and childhood relationships with parents [86]. This study did not show differences between Glasgow, Liverpool and Manchester, although the authors acknowledge that these questions were a “very limited part of the survey and was not aimed at producing a detailed insight into respondents’ childhoods and early years’ experiences”.
6. Contemporary analyses of Scottish longitudinal data showed that the current social, emotional and behavioural development of preschool children in Glasgow (Scotland’s most socio-economically deprived city) is not worse than children elsewhere in Scotland [87].

In summary, some data sources suggest that there are differences in childhood exposure to adversity when comparing areas in Scotland to English comparators. Those differences are most evident in routinely collected data, including rates of imprisonment, domestic violence, problem substance use and numbers of children taken into care. By contrast, data sources suggesting little or no differences tend to rely on informant reports of feelings or behaviours (e.g. from surveys), which are more subject to recall and cultural biases.

### Future research

Currently available data do not allow us to study possible links between relevant exposures (childhood adversity and complex trauma), responses (attachment) and adult health outcomes.

In order to examine the hypothesis that excess mortality in Scotland and especially the West of Scotland relates to childhood experience of attachment and adversity, future studies would require to:

1. Be able to collect accurate measures of childhood adversity, complex trauma and attachment
2. Be able to consider those measures in the context of structural and material factors
3. Be clear about the period of risk exposure and the latency between exposure and outcome in order to identify appropriate age cohorts for comparison
4. Be sensitive to potential selection and responder biases, given the propensity for ACE scores and attachment style to influence recruitment, and the sensitive nature of the questions
5. Be able to adjust for a range of potential confounding variables
6. Investigate these effects with appropriate geographical comparisons.

Each of these considerations brings challenges to designing a research study.

## Conclusions

Scotland has a persistently high mortality rate that is not solely due to the effects of socio-economic deprivation. This “excess” mortality is observed across the whole country, but is greatest in and around the post-industrial conurbation of West Central Scotland. Its causes remain the subject of discussion and debate, despite systematic investigation.

Nonetheless, there is now strong evidence to show that childhood experience of both adversity and attachment exert a significant influence on adult morbidity and mortality. Each of these factors is therefore a plausible influence on the excess mortality observed in Scotland.

We describe in this paper a possible model through which socio-economic factors might influence childhood experiences in a way that not only leads to harm in adulthood, but simultaneously exposes the next generation to similar risks. In this model, childhood adversity leads to damaging metabolic, immune, behavioural and developmental changes, which can be offset or mitigated to some extent by secure attachment experiences.

Inter-generational effects suggested by the model may serve to perpetuate and entrench poorer health in some population groups, especially when those groups are exposed to ongoing adversity.

Thinking about Scotland’s health problems from this perspective links social, environmental and political factors to the health of individuals and groups. Since income, wealth, power, environment and access to services all have the potential to affect childhood adversity and attachment processes, this approach is consistent with a “structural” perspective on health inequalities, which holds that differences in the socioeconomic circumstances, at all stages of the life-course, cause differences in health outcomes” [88].

We present data from a number of sources with which to examine this model. The critical aspect of the hypothesis is that there might be “something different” about childhood experiences in Scotland, and especially West Central Scotland, compared to other areas. Unfortunately, existing data are conflicting. In general, routinely-collected data that signal higher exposure to adversity - such as domestic violence, problem substance use, rates of imprisonment and rates of children being taken into care - indicate an excess of childhood adversity in Scotland. However measures requiring self-report about current or historic aspects of social and family functioning (eg parents reading to the child) tend not to show significant differences.

Existing datasets were not designed to answer these questions, and are subject to a number of difficulties: the samples may not be optimal, the questions asked of participants may not be appropriate, and comparisons across countries and over decades are subject to recall and cultural biases.

The model presented here emphasises the methodological complexities of conducting a study capable of exploring this hypothesis. We hope that this developmental perspective on those challenges will support future work in this important area.

## Abbreviations

ACEs, adverse childhood experiences; DSM-5, diagnostic and statistical manual of mental disorders

## Appendix 1 Measures of childhood adversity in the 1958 National Child Development Study

- Child in care: child has ever been in public/voluntary care services or foster care at age 7, 11 or 16.
- Physical neglect: child appears undernourished/dirty aged 7 or 11, based on information collected by response to teacher to the Bristol Social Adjustment Guide.
- Offenders: child lived in a household where a family member was in prison or probation (aged 11 years) or is in contact with probation service at 7 or 11; the child has ever been to prison or been on probation at 16 years.
- Parental separation: The child has been separated from their father or mother due to death, divorce or separation at 7,11 or 16 years.
- Mental illness: Household has contact with mental health services at ages 7 or 11, family member has mental illnesses at 7 or 11 years.
- Alcohol abuse: Family member has alcohol abuse problem at 7 years (based on the health visitor’s assessment, without questioning the family).

For each positive response to the six categories, the cohort member accumulated one adversity (up to a maximum of six).

## Appendix 2: measures of childhood experience from Taulbut [87]

Information collected included the following: social class of father, low birth weight, mother’s education (or in school after minimum leaving age in the 1958 study), housing tenure, age of mother at birth of child, smoking during pregnancy (after 4 months pregnant in 1958 study), ever breastfed, maternal mental health (1970 study only), Dad helps mum put child to bed (1970 study only), who read to child in last week (frequency mum/dad read to child in 1958 study), Rutter scores, respiratory problems and vocabulary problems.

## References

[1] McCartney G, Walsh D, Whyte B, Collins C (2012). Has Scotland always been the “sick man” of Europe? An observational study from 1855 to 2006. Eur J Public Health.

[2] McCartney G, Collins C, Walsh D, Batty D. Accounting for Scotland’s excess mortality: towards a synthesis. Glasgow Centre for Population Health Glasgow; 2011.

[3] Walsh D, Bendel N, Jones R, Hanlon P (2010). It’s not “just deprivation”: why do equally deprived UK cities experience different health outcomes?. Public Health.

[4] Walsh D, Bendel N, Jones R, Hanlon P. Investigating a Glasgow effect- why do equally deprived UK cities experience different health outcomes? Glasgow Centre for Population Health Glasgow.

[5] Schofield L, Walsh D, Munoz-Arroyo R, McCartney G, Buchanan D, Lawder R, Armstrong M, Dundas R, Leyland AH. Dying younger in Scotland: trends in mortality and deprivation relative to England and Wales, 1981–2011. Health Place. 2016. in press. 10.1016/j.healthplace.2016.05.007 27235691

[6] McCartney G, Collins C, Walsh D, Batty GD (2012). Why the Scots die younger: synthesizing the evidence. Public Health.

[7] Pedace L. Child wellbeing in England, Scotland and Wales: comparisons and variations. London: Family and Parenting Institute, 2008.

[8] Taulbut M, Walsh D. Poverty, parenting and poor health: comparing early years’ experiences in Scotland, England and Three City Regions. Glasgow Centre for Population Health Glasgow; 2013.

[9] Collins C, McCartney G (2011). The impact of neoliberal “Political Attack” on health: the case of the “Scottish Effect”. Int J Heal Serv.

[10] Walsh D, McCartney G, Collins C, Taulbut M, Batty GD. History, politics and vulnerability: explaining excess mortality in Scotland and Glasgow. Glasgow Centre for Population Health Glasgow; 2016. 10.1016/j.puhe.2017.05.016 28697372

[11] Donnelly PD (2010). Explaining the Glasgow effect: could adverse childhood experiences play a role?. Public Health.

[12] Felitti V, Anda R, Nordenberg D (1998). Relationship of childhood abuse and household dysfunction to many of the leading causes of death in adults: the Adverse Childhood Experiences (ACE) study. Am J Prev Med.

[13] Felitti VJ, Anda RF, Lanius RA, Vermetten E, Pain C (2010). The relationship of adverse childhood experiences to adult medical disease, psychiatric disorders and sexual behavior: implications for healthcare. The impact of early life trauma on health and disease: the hidden epidemic.

[14] Kelly-Irving M, Lepage B, Dedieu D, Bartley M, Blane D, Grosclaude P, Lang T, Delpierre C (2013). Adverse childhood experiences and premature all-cause mortality. Eur J Epidemiol.

[15] Bellis M, Lowey H, Leckenby N, Hughes K, Harrison D (2014). Adverse childhood experiences: retrospective study to determine their impact on adult health behaviours and health outcomes in a UK population. J Public Health (Bangkok).

[16] Bellis MA, Hughes K, Leckenby N, Hardcastle KA, Perkins C, Lowey H (2015). Measuring mortality and the burden of adult disease associated with adverse childhood experiences in England: a national survey. J Public Health (Oxf).

[17] Jaffee SR, Caspi A, Moffitt TE, Polo-Tomás M, Taylor A (2007). Individual, family, and neighborhood factors distinguish resilient from non-resilient maltreated children: a cumulative stressors model. Child Abuse Negl.

[18] Sethi D. European report on preventing child maltreatment. World Health Organisation, Copenhagen 2013.

[19] Dube S, Felitti V, Dong M, Chapman D, Giles W, Anda R (2003). Childhood abuse, neglect and household dysfunction and the risk of illicit drug use: the adverse childhood experience study. Pediatrics.

[20] Dube SR, Anda RF, Felitti VJ, Edwards VJ, Croft JB (2002). Adverse childhood experiences and personal alcohol abuse as an adult. Addict Behav.

[21] Bifulco A, Kwon J, Jacobs C, Moran PM, Bunn A, Beer N (2006). Adult attachment style as mediator between childhood neglect/abuse and adult depression and anxiety. Soc Psychiatry Psychiatr Epidemiol.

[22] Difilippo JM, Overholser JC (2002). Depression, adult attachment, and recollections of parental caring during childhood. J Nerv Ment Dis.

[23] Styron T, Janoff-Bulman R (1997). Childhood attachment and abuse: long-term effects on adult attachment, depression, and conflict resolution. Child Abuse Negl.

[24] Afifi TO, Enns MW, Cox BJ, Asmundson GJG, Stein MB, Sareen J (2008). Population attributable fractions of psychiatric disorders and suicide ideation and attempts associated with adverse childhood experiences. Am J Public Health.

[25] Copeland WE, Wolke D, Angold A, Costello EJ (2013). Adult psychiatric outcomes of bullying and being bullied by peers in childhood and adolescence. JAMA Psychiatry.

[26] McLaughlin K, Green JG, Gruber MJ, Sampson N, Zaslavsky a M, Kessler RC (2010). Childhood adversities and adult psychopathology in the National Comorbidity Survey Replication (NCS-R) III: associations with functional impairment related to DSM-IV disorders. Psychol Med.

[27] Burke NJ, Hellman JL, Scott BG, Weems CF, Carrion VG (2011). The impact of adverse childhood experiences on an urban pediatric population. Child Abuse Negl.

[28] Greenwald R (2013). The role of trauma in conduct disorder. J Aggress Maltreat Trauma.

[29] Smith C, Thornberry TP (1995). The relationship between childhood maltreatment and adolescent involvement in delinquency. Criminology.

[30] Krueger RF, Caspi A, Moffitt TE (2000). Epidemiological personology: the unifying role of personality in population-based research on problem behaviors. J Pers.

[31] Luthar S, Cicchetti D, Becker B (2003). The construct of resilience: a critical evaluation and guidelines for future work. Child Dev.

[32] Shonkoff JP, Garner AS (2012). The lifelong effects of early childhood adversity and toxic stress. Pediatrics.

[33] Herman JL (1992). Trauma and recovery.

[34] Courtois C, Ford J (2009). Treating complex traumatic stress disorders: an evidence based guide.

[35] BC Provincial Mental Health and Substance Use PlanningCouncil (2013). Trauma-informed practice guide.

[36] Kessler RC, Ustün TB (2004). The World Mental Health (WMH) Survey Initiative Version of the World Health Organization (WHO) Composite International Diagnostic Interview (CIDI). Int J Methods Psychiatr Res.

[37] Koenen KC, Roberts AL, Stone DM, Dunn EC. The epidemiology of early childhood trauma. In: Lanius RA, Vermetten E, Pain C, editors. The Impact of Early Life Trauma on Health and Disease: The Hidden Epidemic. Cambridge University Press, Cambridge; 2010. p. 13–24.

[38] Torchalla I, Nosen L, Rostam H, Allen P (2012). Integrated treatment programs for individuals with concurrent substance use disorders and trauma experiences: a systematic review and meta-analysis. J Subst Abuse Treat.

[39] Hammersley R, Dalgarno P (2013). Trauma and recovery amongst people who have injected drugs within the past 5 years.

[40] American Psychiatric Association. Diagnostic and statistical manual of mental disorders, Fifth Edition (DSM-5). VA: American Psychiatric Publishing, 2013.

[41] Bowlby J (1979). The making and breaking of affectional bonds.

[42] Thompson R (2000). The legacy of early attachments. Child Dev.

[43] Berry K, Danquah AN, Wallin D (2013). Introduction to attachment. Attachment theory in adult mental health.

[44] Ciechanowski P, Sullivan M, Jensen M, Romano J, Summers H (2003). The relationship of attachment style to depression, catastrophizing and health care utilization in patients with chronic pain. Pain.

[45] Ciechanowski PS, Walker EA, Katon WJ, Russo JE (2002). Attachment theory: a model for health care utilization and somatization. Psychosom Med.

[46] Ainsworth MD (1985). Attachments across the life span. Bull N Y Acad Med.

[47] Karen R. Attachment in adulthood. In: Becoming Attached: First Relationships and How They Shape Our Capacity to Love. New York: Oxford University Press. 1998:379–373.

[48] Scroufe LA. Infant–caregiver attachment and patterns of adaptation in pre-school: The roots of maladaptation and competence. In: Perlmutter M. Hillsdale, editor. Minnesota Symposium in Child Psychology. Hillsdale, NJ: Erlbaum Associates; 1983:41–81.

[49] Hazan C, Shaver P. Attachment as an organizational framework for research on close relationships. Psychological Inquiry. 1994;5:1–22.

[50] Shafto P, Eaves B, Navarro DJ, Perfors A (2012). Epistemic trust: modeling children’s reasoning about others’ knowledge and intent. Dev Sci.

[51] Eaves BS, Shafto P (2012). Unifying pedagogical reasoning and epistemic trust. Adv Child Dev Behav.

[52] Fonagy P, Steele M, Moran G, Steele H, Higgitt A (1993). Measuring the ghost in the nursery: an empirical study of the relation between parents’ mental representations of childhood experiences and their infants’ security of attachment. J Am Psychoanal Assoc.

[53] Bifulco A, Moran PM, Ball C, Jacobs C, Baines R, Bunn A, Cavagin J (2002). Childhood adversity, parental vulnerability and disorder: examining inter-generational transmission of risk. J Child Psychol Psychiatry.

[54] Bifulco A, Moran P, Jacobs C, Bunn A (2009). Problem partners and parenting: exploring linkages with maternal insecure attachment style and adolescent offspring internalizing disorder. Attach Hum Dev.

[55] Bowlby J (1998). Attachment and loss (Volume 3).

[56] Mikulincer M, Gillath O, Shaver PR (2002). Activation of the attachment system in adulthood: threat-related primes increase the accessibility of mental representations of attachment figures. J Pers Soc Psychol.

[57] Riggs SA, Vosvick M, Stallings S (2007). Attachment style, stigma and psychological distress among HIV+ adults. J Health Psychol.

[58] Hunter JJ, Maunder RG (2001). Using attachment theory to understand illness behavior. Gen Hosp Psychiatry.

[59] Bifulco A, Moran PM, Ball C, Lillie A (2002). Adult attachment style. II: Its relationship to psychosocial depressive-vulnerability. Soc Psychiatry Psychiatr Epidemiol.

[60] Cohen O, Birnbaum GE, Meyuchas R, Levinger Z, Florian V, Mikulincer M (2005). Attachment orientations and spouse support in adults with type 2 diabetes. Psychol Health Med.

[61] Bifulco A, Thomas G. Understanding adult attachment in family relationships: research, assessment and intervention Routledge, London; 2012.

[62] McEwen BS (2000). Allostasis and allostatic load: implications for neuropsychopharmacology. Neuropsychopharmacology.

[63] Lupien SJ, McEwen BS, Gunnar MR, Heim C (2009). Effects of stress throughout the lifespan on the brain, behaviour and cognition. Nat Rev Neurosci.

[64] Danese A, Moffitt TE, Harrington H, Milne BJ, Polanczyk G, Pariante CM, Poulton R, Caspi A (2009). Adverse childhood experiences and adult risk factors for age-related disease: depression, inflammation, and clustering of metabolic risk markers. Arch Pediatr Adolesc Med.

[65] Briggs RD, Silver EJ, Krug LM, Mason ZS, Schrag RDA, Chinitz S, Racine AD. Healthy Steps as a moderator: the impact of maternal trauma on child social-emotional development. Clinical Practice in Pediatric Psychology. 2014;2:166-175

[66] Robertson T, Popham F, Benzeval M (2014). Socioeconomic position across the lifecourse & allostatic load: data from the West of Scotland Twenty-07 cohort study. BMC Public Health.

[67] National Scientific Council on the Developing Child (2012). The science of neglect-the persistent absence of responsive care disrupts the developing brain.

[68] Danese A, Moffitt TE, Harrington H, Milne BJ, Polanczyk G, Pariante CM, Poulton R, Caspi A (2010). Adverse childhood experiences and adult risk factors for age-related disease. Arch Pediatr Adolesc Med.

[69] Clark C, Caldwell T, Power C, Stansfeld SA (2010). Does the influence of childhood adversity on psychopathology persist across the lifecourse? A 45-year prospective epidemiologic study. Ann Epidemiol.

[70] Serretti A, Fabbri C (2013). Shared genetics among major psychiatric disorders. Lancet.

[71] Capaldi DM, Patterson GR (1991). Relation of parental transitions to boys’ adjustment problems: I. A linear hypothesis: II. Mothers at risk for transitions and unskilled parenting. Dev Psychol.

[72] Raikes HA, Thompson RA (2005). Links between contextual risk and attachment: models of influence. Appl Dev Psychol.

[73] Investigators FLP (2013). Cumulative risk and its relation to parenting and child outcomes at 36 months. Monogr Soc Res Child Dev.

[74] Hobcraft J, Kiernan K. Predictive factors from age 3 and infancy for poor child outcomes at age 5 relating to children’s development, behaviour and health: evidence from the millennium cohort study. University of York, York; 2010.

[75] Murphy A, Steele M, Dube SR, Bate J, Bonuck K, Meissner P, Goldman H, Steele H (2014). Adverse Childhood Experiences (ACEs) questionnaire and Adult Attachment Interview (AAI): implications for parent child relationships. Child Abuse Negl.

[76] Gervai J (2009). Environmental and genetic influences on early attachment. Child Adolesc Psychiatry Ment Health.

[77] Waters E, Merrick S, Treboux D, Crowell J, Albersheim L (2000). Attachment security in infancy and early adulthood: a twenty-year longitudinal study. Child Dev.

[78] Gruenewald TL, Karlamangla AS, Hu P, Stein-Merkin S, Crandall C, Koretz B, Seeman TE (2012). History of socioeconomic disadvantage and allostatic load in later life. Soc Sci Med.

[79] Plsek PE, Greenhalgh T (2001). Complexity science: the challenge of complexity in health care. BMJ.

[80] Hallqvist J, Lynch J, Bartley M, Lang T, Blane D (2004). Can we disentangle life course processes of accumulation, critical period and social mobility? An analysis of disadvantaged socio-economic positions and myocardial infarction in the Stockholm Heart Epidemiology Program. Soc Sci Med.

[81] MacNicol J (1987). In pursuit of the underclass. J Soc Policy.

[82] Welshman J. Underclass: A History of the Excluded Since 1880. 2nd Revision. London: Bloomsbury Academic, 2013.

[83] van IJzendoorn MH, Bakermans-Kranenburg MJ (1996). Attachment representations in mothers, fathers, adolescents, and clinical groups: a meta-analytic search for normative data. J Consult Clin Psychol.

[84] Mok PLH, Leyland AH, Kapur N, Windfuhr K, Appleby L, Platt S, Webb RT (2013). Why does Scotland have a higher suicide rate than England? An area-level investigation of health and social factors. J Epidemiol Community Health.

[85] Taulbut M, Walsh D, O’Dowd J (2014). Comparing early years and childhood experiences and outcomes in Scotland, England and three city-regions: a plausible explanation for Scottish “excess” mortality?. BMC Pediatr.

[86] Walsh D, McCartney G, McCullough S, van der Pol M, Buchanan D, Jones R. Exploring potential reasons for Glasgow’s “excess” mortality: results of a three-city survey of Glasgow, Liverpool and Manchester. Glasgow Centre for Population Health, Glasgow; 2013.

[87] Marryat L, Thompson L, Minnis H, Wilson P (2015). Exploring the social, emotional and behavioural development of preschool children: is Glasgow different?. Int J Equity Health.

[88] McCartney G, Collins C, Mackenzie M (2013). What (or who) causes health inequalities: theories, evidence and implications?. Health Policy.

